# Complete or overcompensatory thermal acclimation of leaf dark respiration in African tropical trees

**DOI:** 10.1111/nph.17038

**Published:** 2020-12-06

**Authors:** Myriam Mujawamariya, Maria Wittemann, Aloysie Manishimwe, Bonaventure Ntirugulirwa, Etienne Zibera, Donat Nsabimana, Göran Wallin, Johan Uddling, Mirindi Eric Dusenge

**Affiliations:** ^1^ Department of Biology University of Rwanda University Avenue PO Box 117 Huye Rwanda; ^2^ Department of Biological and Environmental Sciences University of Gothenburg PO Box 461 Gothenburg SE‐405 30 Sweden; ^3^ Rwanda Agriculture and Animal Development Board PO Box 5016 Kigali Rwanda; ^4^ School of Forestry and Biodiversity and Biological Sciences University of Rwanda Busogo Rwanda

**Keywords:** drought, elevation gradient, montane, photosynthesis, successional groups, warming

## Abstract

- Tropical climates are getting warmer, with pronounced dry periods in large areas. The productivity and climate feedbacks of future tropical forests depend on the ability of trees to acclimate their physiological processes, such as leaf dark respiration (*R*_d_), to these new conditions. However, knowledge on this is currently limited due to data scarcity.
- We studied the impact of growth temperature on *R*_d_ and its dependency on net photosynthesis (*A*_n_), leaf nitrogen (N) and phosphorus (P) contents, and leaf mass per unit area (LMA) in 16 early‐successional (ES) and late‐successional (LS) tropical tree species in multispecies plantations along an elevation gradient (Rwanda TREE project). Moreover, we explored the effect of drought on *R*_d_ in one ES and one LS species.
- Leaf *R*_d_ at 20°C decreased at warmer sites, regardless if it was expressed per unit leaf area, mass, N or P. This acclimation resulted in an 8% and a 28% decrease in *R*_d_ at prevailing nighttime temperatures in trees at the intermediate and warmest sites, respectively. Moreover, drought reduced *R*_d_, particularly in the ES species and at the coolest site.
- Thermal acclimation of *R*_d_ is complete or overcompensatory and independent of changes in leaf nutrients or LMA in African tropical trees.

## Introduction

Global mean temperature is predicted to rise by 1–4°C by 2100 as a consequence of increasing glasshouse gas concentrations, and the tropics could warm by 1–3°C (IPCC, 2013). In addition, more frequent and severe drought events are predicted over the 21st century in tropical regions (Malhi *et al*., 2008; Chadwick *et al*., 2015). Terrestrial vegetation plays an important role in regulating climate through biogeochemical and biophysical processes (Bonan, 2008; Arneth *et al*., 2010). Through photosynthesis, terrestrial vegetation absorbs *c*. 123 Gt of C from the atmosphere each year (Beer *et al*., 2010). However, about half of this fixed carbon is released back to the atmosphere through autotrophic respiration (Ciais *et al*., 2013), and around half of the autotrophic respiration comes from leaves (Atkin *et al*., 2007; Campioli *et al*., 2016). Anthropogenic CO_2_ emissions currently release *c*. 11 Gt C per year to the atmosphere (Friedlingstein *et al*., 2019), representing one‐sixth of autotrophic respiration. This implies that a rather small fractional change in plant respiration due to climate change could have large consequences on the rate at which the atmospheric CO_2_ concentration is increasing (Cox *et al*., 2000; Anderegg *et al*., 2015; Liu *et al*., 2017).

African tropical forests contribute significantly to the terrestrial carbon sequestration (Nyirambangutse *et al*., 2017; Sullivan *et al*., 2020). They currently take up *c*. 0.63 tonnes of C per hectare per year but, due to increasing temperature and drought, this carbon sink strength is predicted to decrease by 14% by 2030 (Hubau *et al*., 2020). The decline in carbon gain and sink may occur if respiration (autotrophic and/or heterotrophic) increases or if net photosynthesis decreases, or if both scenarios occur simultaneously in hot and dry conditions. A recent study on temperature sensitivity of tropical forests across a pantropical network of permanent plots showed that declines in carbon gain are driven by high daytime rather than high nighttime temperatures (Sullivan *et al*., 2020). This was suggested to be due to decreases in photosynthesis while respiration may readily acclimate to warming. However, our knowledge regarding the acclimation potential of these two physiological processes to warming and drought in tropical trees is currently very limited (Huntingford *et al*., 2013; Smith & Dukes, 2013; Mercado *et al*., 2018). Strong warming sensitivity of tropical trees has also been demonstrated by recent studies along tropical elevation gradients, reporting significant shifts towards lower relative abundances of higher elevation (i.e. cooler adapted) tree species over time during the last three decades (Duque *et al*., 2015; Fadrique *et al*., 2018).

Leaf respiration in darkness (*R*_d_) produces ATP, reducing equivalents and carbon skeletons needed for plant growth and cellular maintenance, but also releases CO_2_ as a by‐product (reviewed in O’Leary *et al*., 2019). Leaf *R*_d_ is temperature dependent and in the short‐term (minutes to hours) it increases exponentially with increasing temperature (Atkin & Tjoelker, 2003; Atkin *et al*., 2005; O’Sullivan *et al*., 2013; Reich *et al*., 2016). However, when plants are grown in warmer temperatures, *R*_d_ usually acclimates such that *R*_d_ measured at a common leaf temperature is lower in warm‐grown plants compared with their cool‐grown counterparts (Atkin & Tjoelker, 2003; Atkin *et al*., 2005; Tjoelker *et al*., 2009; Slot & Kitajima, 2015; Reich *et al*., 2016). This thermal acclimation of *R*_d_ is usually partial rather than complete, such that *R*_d_ measured at the respective growth temperature is still higher in warm‐grown trees compared with their cool‐grown counterparts (Slot & Kitajima, 2015; Vanderwel *et al*., 2015; Reich *et al*., 2016; Smith & Dukes, 2017).

Partial thermal acclimation of leaf *R*_d_ is in agreement with a recently proposed theory on optimal acclimation of photosynthetic capacity (Wang *et al*., 2020). Based on the theory of optimal photosynthetic capacity, the maximum carboxylation capacity of RuBisCo (*V*_cmax_) is expected to decrease in warm‐grown plants, as enzymes can still maintain a high activity at a lower content when operating at higher temperatures (Smith & Keenan, 2020). The theory further suggests that thermal acclimation of leaf respiration should follow closely that of photosynthetic capacity and *V*_cmax_. As RuBisCo accounts for a large proportion of the total leaf protein content, the respiratory energy required for protein maintenance (the largest determinant of variation in leaf *R*_d_) should be linked to the activity and content of this important enzyme. The theory is supported by a global observational dataset showing that *R*_d_ is strongly and positively related to *V*_cmax_ (Atkin *et al*. (2015). Moreover, another meta‐analysis demonstrated decreases in both *V*_cmax_ and *R*_d_ (at a common measurement temperature) in warm‐grown plants, suggesting that thermal acclimation of leaf *R*_d_ may be strongly driven by that of *V*_cmax_ (Wang *et al*., 2020). Both studies indicated that the thermal acclimation in leaf *R*_d_ does not completely offset increases in leaf *R*_d_ when measured at a higher growth temperature; i.e. *R*_d_ thermal acclimation is partial, not complete or ‘homeostasis’. Although this seem to be the most common type of response, the prediction of partial thermal acclimation of *R*_d_ by the optimality model (Wang *et al*., 2020) is at odds with observations of complete acclimation (Cheesman & Winter, 2013a; Slot & Kitajima, 2015; Slot & Winter, 2018) or complete lack of thermal acclimation of *R*_d_ (Crous *et al*., 2017b; Kurepin *et al*., 2018) in some studies.

Thermal acclimation of *R*_d_ has been studied in some tropical tree species, either grown in controlled‐environment chambers (Cheesman & Winter, 2013a,b; Drake *et al*., 2015; Scafaro *et al*., 2017; Slot & Winter, 2017, 2018; Smith & Dukes, 2017; Zhu *et al*., 2021) or with warming applied to individual branches for short periods (Slot *et al*., 2014). In most of these studies some acclimation occurred, but the magnitude to which thermal acclimation of *R*_d_ reduces respiratory CO_2_ release in warm‐grown tropical tree species under realistic field settings is still highly uncertain. Is it partial, lacking or perhaps even complete (i.e. leading to homeostasis)? Global meta‐analyses (Slot & Kitajima, 2015; Dusenge *et al*., 2019) have shown that partial acclimation is most common, but they are dominated by data from temperate species and cannot be used to draw firm conclusions regarding acclimation potential of tropical trees.

Respiratory metabolism comprises complex and coordinated biochemical processes that take place in different organelles in the cell (Plaxton & Podestá, 2006; O’Leary *et al*., 2019). There is still no clear consensus of the exact physiological and biochemical mechanisms underlying thermal acclimation of *R*_d_ (Dusenge *et al*., 2019). Leaf *R*_d_ is coupled with photosynthesis as it provides substrates for respiration (Hoefnagel *et al*., 1998; Atkin & Tjoelker, 2003; Atkin *et al*., 2005, 2015; Rowland *et al*., 2017; Crous *et al*., 2017a; O’Leary *et al*., 2019), and some studies have shown that thermal acclimation of these two processes is coordinated (Dusenge *et al*., 2019; Wang *et al*., 2020; but see Rashid *et al*., 2020). Moreover, interspecific variation in *R*_d_ may correlate with leaf nutrient content, notably nitrogen (N) (Ryan, 1995; Reich *et al*., 1998; Atkin & Tjoelker, 2003; Atkin *et al*., 2005, 2015; Wright *et al*., 2006; Rowland *et al*., 2017; Crous *et al*., 2017a) and phosphorus (P) (Theodorou & Plaxton, 1993; Meir *et al*., 2001; Wang *et al*., 2015; Rowland *et al*., 2017; Crous *et al*., 2017a), as these two macro‐elements make up a large proportion of enzymes, ATP, reducing equivalents, and sugar phosphates involved in respiratory metabolism (Plaxton & Podestá, 2006; Taiz *et al*., 2014; O’Leary *et al*., 2019). However, while some previous studies have shown that thermal acclimation of *R*_d_ was underpinned by concurrent decreases in leaf nitrogen (N) (Tjoelker *et al*., 1999; Crous *et al*., 2017b; Ahmad Rashid *et al*., 2020; Dusenge *et al*., 2020), the possible link to leaf phosphorus (P) has been less studied. Andean field studies have reported higher leaf *R*_d_ (van de Weg *et al*., 2012) and leaf P content (Fyllas *et al*., 2017) in montane compared with lowland forests, while a study on forests in Peru, French Guyana and Australia found the highest *R*_d_ at the site with lowest fertility and leaf P (Rowland *et al*., 2017). Finally, interspecific variation in leaf *R*_d_ has been shown to positively correlate with leaf mass per unit area (LMA) (Reich *et al*., 1998; Wright *et al*., 2006; Crous *et al*., 2017a), but its role in thermal acclimation of *R*_d_ is uncertain.

Plant functional groups differ inherently in their basal rates of respiration (Atkin *et al*., 2015; Crous *et al*., 2017a), and these differences may be strongly linked to their contrasting ecological growth strategies (Grime, 1977; Wright *et al*., 2004). In tropical forests, early‐successional (ES; shade intolerant and fast‐growing) and late‐successional (LS; shade tolerant and slow‐growing) tree species exhibit strongly contrasting growth strategies (Nyirambangutse *et al*., 2017; Ntawuhiganayo *et al*., 2020). Some studies with few species have indicated that these two groups might respond differently to warming, with LS species sometimes showing negative responses of growth and photosynthesis to warming while ES species are generally less affected (Cheesman & Winter, 2013b; Slot & Winter, 2018). It is currently unclear if species of different successional strategies differ in their ability to acclimate *R*_d_ to growth temperature.

Drought commonly reduces photosynthesis through decreased stomatal conductance and photosynthetic capacity (Flexas *et al*., 2001; Gulías *et al*., 2002; Slot *et al*., 2008; Damour *et al*., 2008, 2009; Atkin & Macherel, 2009; Ayub *et al*., 2011; Mujawamariya *et al*., 2018; but see Rowland *et al*., 2015b). In many cases this results in decreased *R*_d_, most likely as a consequence of decreased carbohydrate supply from photosynthesis to respiratory metabolism (Griffin *et al*., 2002; Whitehead *et al*., 2004; Galmés *et al*., 2007; Atkin & Macherel, 2009; Ayub *et al*., 2011; Crous *et al*., 2011; O'Leary *et al*., 2017, 2019). In addition, when photosynthesis is decreased during drought, the energy cost of sucrose production for phloem loading also declines (Lawlor & Fock, 1977). Thus, reduced leaf *R*_d_ in response to drought may be linked to both reduced sugar substrates availability and energy required for their transport (Atkin & Macherel, 2009). However, in some studies, *R*_d_ was instead increased under dry conditions (Miranda *et al*., 2005; Slot *et al*., 2008; Metcalfe *et al*., 2010; Rowland *et al*., 2015b). This response may serve to provide energy for cellular maintenance (Atkin & Macherel, 2009), particularly for hydraulic repair needed in drought conditions (Brodersen & McElrone, 2013; Rowland *et al*., 2015a). In some studies, leaf *R*_d_ was unaltered in response to drought (Galmés *et al*., 2007; Atkin & Macherel, 2009; Gimeno *et al*., 2010). African tropical forests experience less extreme droughts compared with Amazon forests, and are also suggested to be less sensitive to drought (Hubau *et al*., 2020). However, no study to date has investigated the impacts of drought on *R*_d_ in African tropical tree species.

We studied the thermal acclimation and interspecific variation in leaf *R*_d_ among 10 ES and six LS tropical montane or highland tree species planted at three different sites along an elevation gradient differing in mean annual temperature, being 4.8°C and 5.4°C higher for the mid‐elevation Rubona and low‐elevation Makera sites, respectively, compared with the high‐elevation Sigira site. We also investigated the drought response of leaf *R*_d_ in one ES and LS tree species grown at the same sites. The following hypotheses were tested:

1. Leaf *R*_d_ acclimates to growth temperature (i.e. to be reduced in warm‐grown trees at a common measurement temperature), and this acclimation is linked to concurrent changes in other leaf traits (leaf N, P, LMA, or photosynthesis).
2. The thermal acclimation of *R*_d_ is partial, i.e. not strong enough to prevent increased *R*_d_ at prevailing nighttime temperatures under warmer growth conditions.
3. Given strong control of sugar substrate availability on *R*_d_, leaf *R*_d_ is lower during late‐dry season compared with early‐dry season, particularly at warmer sites with more severe drought.
4. ES species exhibit stronger thermal acclimation of leaf *R*_d_ compared with LS species.
5. Net photosynthesis and leaf N are stronger predictors of interspecific variation and thermal acclimation of leaf *R*_d_ compared with leaf P or LMA.

## Materials and Methods

### Experimental sites

The study was conducted within the Rwanda TREE (Tropical Elevational gradient Experiment) project which consist of experimental tree plantations established at three different sites along an elevation gradient in Rwanda, Central Africa (Table 1). These sites are within *c*. 250 km distance, and they exhibit large variations in elevation (1300–2400 m above sea level (asl)) and climate (Table 1). The plantation sites belong to the Rwanda Agriculture and Animal Resources Development Board. The high‐elevation site (2400 m asl) is located at Sigira in Nyamagabe district (hereafter called Sigira) in close proximity to the plantation buffer zone surrounding Nyungwe National Park, a tropical montane rainforest in southwestern part of Rwanda. The Sigira site is also considered as the control site in this experiment, as most species used in this experiment naturally grow in the neighbouring montane rainforest (Nyirambangutse *et al*., 2017). The mid‐elevation site (1600 m asl) is located at Rubona in Huye district (hereafter called Rubona), also in the southwestern part of Rwanda, *c*. 50 km south‐east from Sigira site. The low‐elevation site (1300 m asl) is located at Ibanda‐Makera in Kirehe district (hereafter called Makera) in the eastern part of Rwanda, *c*. 250 km east of Sigira and near to the border with Tanzania.

**Table 1:** Study site environmental conditions for the period of February 2018 to January 2020 and soil water content for the period of August 2018 to January 2020 (first part of the table) and for October 2018 (second part of the table), as well as soil water content at the start and end of the June–August dry season (third part of the table).

		Sites	
	Sigira	Rubona	Makera
Potential vegetation type	Montane rainforest	Transitional rainforest	Evergreen and semi‐evergreen bushland and thicket
Elevation (m asl)	2400	1600	1300
Latitude	2°30′54″S	2°28′30″S	2°6′31″S
Longitude	29°23′44″E	29°46′49″E	30°51′16″E
MAT (°C)	15.2	20.0	20.6
MAP (mm)	2100	1700	1100
Mean PPFD, day (µmol m^−2^ s^−1^)	611	770	729
SWC (m^3^ m^−3^)	0.29	0.19	0.20
*T* day (°C)	17.1	22.6	24.2
*T* night (°C)	13.2	17.5	16.6
*T* 1%ile (°C)	10.9	13.3	10.5
*T* 99%ile (°C)	23.1	28.5	31.2
Daytime vapour pressure deficit of the air (kPa)	0.88	1.37	1.49
Environmental conditions during 30 d before measurements in November 2018			
*T* day (°C)	17.1	22.6	24.2
*T* night (°C)	13.5	17.8	17.1
*T* 1‰ (°C)	11.2	14.4	12.5
*T* 99‰ (°C)	23.1	28.3	31.1
Mean PPFD, day (µmol m^−2^ s^−1^)	598	839	759
SWC (m^3^ m^−3^)	0.26	0.20	0.19
SWC during at the start and end of dry period			
SWC, May/June 2019 (m^3^ m^−3^)	0.35	0.19	0.22
SWC, August 2019 (m^3^ m^−3^)	0.24	0.15	0.13

MAP, mean annual precipitation; MAT, mean annual temperature; PPFD, photosynthetic photon flux density; SWC, soil water content; *T* day, day temperature; *T* 99‰, temperature 99 percentile; *T* night, night temperature, *T* 1‰, temperature 1 percentile.

As a consequence of their location at different elevations, these sites differ in annual mean air temperature (Table 1), with the high‐elevation Sigira, being on average the coolest site (15.2°C), while mid‐elevation Rubona (20.0°C) and low‐elevation Makera (20.6°C) were *c.* 5°C warmer during the February 2018 to January 2020 period. Differences in average weekly maximum air temperatures were considerably larger: 23.1, 28.5 and 31.2°C for Sigira, Rubona and Makera, respectively. Nights were actually somewhat cooler at the low‐elevation Makera site (16.6°C) compared with at intermediate elevation Rubona (17.5°C), again with highest elevation Sigira site being coolest (13.2°C). The sites also differed substantially in annual precipitation, decreasing progressively from Sigira (*c*. 2100 mm) to Rubona (*c*. 1700 mm) and Makera (*c*. 1100 mm; Table 1). However, the relative distribution of precipitation over the year is similar at all sites, with highest rainfall in March–May and a dry period in June–August. Furthermore, the soil type differs across the three sites. The soil texture is clay to clay loam at Sigira, sandy clay to sandy clay loam at Rubona and sandy clay loam to clay loam at Makera. The three sites occur in different vegetation zones: montane rainforest (Sigira), transitional rain forest (Rubona), and evergreen and semi‐evergreen bushland and thicket (Table 1; Kindt *et al*., 2014). These contrasts in air temperature and precipitation across sites offers an excellent opportunity to evaluate the impact of climate change on tropical tree species.

### Experimental design and plant material

At each site, the plantation is 50 m wide and 102.5 m long with 18 plots of 15 × 15 m each and spaced by 2.5 m. In each plot, plants are spaced by 1.5 × 1.5, allowing 100 plants belonging to 20 different evergreen tropical montane or highland tree species with a replication of five plants. The position of plants in each plot was randomised across species. Seeds or cuttings of each tree species were collected from either montane rainforest or transitional rainforest locations and propagated in poly‐pots in a nursery at the mid‐elevation site (Rubona). Plants were transplanted, after 6–12 months in the nursery and having a height of *c*. 10–75 cm (depending on species), into the soil at the three sites during December 2017 to January 2018. All plants were irrigated equally during the first dry periods in 2018 and early 2019, to allow successful plant establishment. However, the plots studied here were left without irrigation during the main dry season in 2019, with plants therefore being subject to natural seasonal drought from June in that year. The present study used 10 ES and six LS species (Table 2), with equal representation of montane rainforest and transitional rainforest species in each group.

**Table 2:** Tree species and their successional groups.

Scientific names	Author	Family
Early‐successional species		
*Bridelia brideliifolia*	(Pax.) Fedde	Phyllanthaceae
*Bridelia micrantha*	(Hochst.) Baill.	Phyllanthaceae
*Croton megalocarpus*	Hutch.	Euphorbiaceae
*Dombeya rotundifolia*	(Hochst.) Planchon	Malvaceae
*Harungana madagascariensis*	Lam. ex Poiret	Hypericaceae
*Harungana montana*	Spirlet	Hypericaceae
*Macaranga kilimandscharica*	Pax	Euphorbiaceae
*Maesa lanceolata*	Forsk.	Primulaceae
*Markhamia lutea*	(Benth.) K.Schum.	Bignoniaceae
*Polyscias fulva*	(Hiern) Harms	Araliaceae
Late‐successional species		
*Carapa grandiflora*	Sprague	Meliaceae
*Chrysophyllum gorungosanum*	Engl.	Sapotaceae
*Entandrophragma excelsum*	(Dawe & Sprague) Sprague	Meliaceae
*Ficus thonninghii*	Blume	Moraceae
*Prunus africana*	(Hook.f.) Kalkman	Rosaceae
*Syzygium guineense*	(Willd.) DC.	Myrtaceae

### Respiration measurements

Leaf dark respiration (*R*_d_) measurements were conducted in three different campaigns. During the first campaign, the acclimation of *R*_d_ at 20°C to increased growth temperature was investigated in 16 species. To ensure that the determination of thermal acclimation of *R*_d_ was not confounded by site differences in soil water availability, this measurement campaign was conducted in November of 2018, which falls within the annual short rainy season. The precipitation amount during October–November 2018 was 360, 235 and 248 mm for Sigira, Rubona and Makera, respectively. Moreover, the soil water content in November 2018 was close to the site‐specific field capacity, which likely occurs towards the end of the heavy rainy season during March to May (Supporting information Fig. S5; precipitation in March–May 2018 was 980, 832 and 474 mm at Sigira, Rubona and Makera, respectively). During this first campaign, three to five healthy trees of each species and from each site were randomly selected and one fully expanded (but not senescing) leaf per tree was measured. Trees were 20–100, 20–150 and 20–220 cm tall, for Sigira, Rubona and Makera, respectively, when measurements were conducted in November 2018.

The second and the third campaigns were conducted in early‐dry (May–June of 2019) and late‐dry seasons (August of 2019), respectively, and the main purpose of these two campaigns was to assess the seasonal drought effect on foliar respiration. However, contrary to the first campaign, during these two campaigns only two Afromontane rainforest tree species, *Carapa grandiflora* (a LS species) and *Polyscias fulva* (an ES species), were measured. For both species, five to six trees were measured in Rubona and Makera, while nine trees for both species were measured in Sigira. All trees were randomly selected. Each measured branch during early‐dry season campaign was labelled for subsequent measurements during late‐dry season campaign, using a neighbouring leaf to the one measured in early‐dry season. The drought effect was quantified by comparing *R*_d_ values between the two campaigns.

*R_d_* was determined by gas exchange measurements after the sunset between 18:30 and 22:30 at all sites using two portable photosynthesis systems (one LI‐6400 and one LI‐6400‐XT, both with 2 × 3 cm leaf cuvette; Li‐Cor Inc., Lincoln, NE, USA). Although gross leaf efflux of CO_2_ may include some CO_2_ from xylem, this contribution is likely to be small in the dark evening when rates of transpiration are low (Stutz *et al*., 2017). For all measurements, the following settings were used: air flow rate was 250 µmol s^−1^, CO_2_ concentration of air entering the leaf chamber was set at 410 µmol mol^−1^, chamber block temperature was set at 20°C, and the light source was turned off. Before measuring each tree, an empty chamber log was taken in order to allow corrections for any potential leaks in the leaf chamber. Leaf temperatures during measurements varied between 18 and 21°C but were adjusted to an exact leaf temperature of 20°C using Eqn 1 below.

### Estimation of leaf R_d_ at nighttime growth temperature

During the first campaign, we were unable to also measure *R*_d_ at nighttime growth temperature (*R*_dTg_), thus we estimated it from *R*_d_ at 20°C, a fixed Q_10_ (i.e. the quotient of increase in *R*_d_ for a 10°C rise in leaf temperature) and the average nighttime air temperature of the month (October) preceding the measurements. The following Eqn 1 was used (Heskel *et al*., 2016):

(Eqn 1) $$R=R_{d20}*Q_{10}^{\frac{T_{g}‐20}{10}}$$

where *T*_g_ is the average nighttime air temperature of the month preceding the measurement campaign (i.e. October 2018). A *Q*_10_ value of 2.3 was used, based on previous published studies on tropical tree species (Atkin & Tjoelker, 2003; Weerasinghe *et al*., 2014; Slot & Winter, 2017). As *Q*_10_ has also been shown to decrease with increasing growth temperature as an acclimation response to warming (e.g. Atkin & Tjoelker, 2003; Slot & Winter, 2017), we performed a sensitivity analysis using different *Q*_10_ values, one for each site calculated from an Eqn 2 given in Atkin & Tjoelker (2003), and we examined the extent to which our estimates of *R*_d_ at nighttime growth temperature based on a fixed Q_10_ of 2.3 would change.

(Eqn 2) $$Q_{10}=3.09‐0.043T$$

where *T* represent a chosen temperature in °C. Using growth temperature dependent *Q*_10_ values for each site did not change any of our conclusions (see details below in the Results section).

### Net photosynthesis measurements

During the November 2018 field campaign, light saturated net photosynthesis was measured on the same leaves measured for *R*_d_. Photosynthesis measurements were made before *R*_d_ measurements, between 09:00 h and 16:30 h on the same day. The temperature in the LI6400 leaf cuvette was controlled by the setting the block temperature to 25°C (with leaf temperatures varying between 25 and 29°C at the high‐elevation site, and between 26 and 31°C at the two lower elevation sites). Other LI6400 settings were: air flow rate of 400 µmol s^−1^, CO_2_ concentration of air entering the leaf chamber at 415 µmol mol^−1^ and the photosynthetic photon flux density (PPFD) at 1800 µmol m^−2^ s^−1^.

### Chemical and morphological traits

For each leaf measured for gas exchange three discs of known diameter were collected for subsequent determinations of LMA and leaf N concentration. Furthermore, leaf P concentration was also analysed in 36 leaves of each species and site sampled during a period close to the November 2018 *R*_d_ measurement campaign, and leaf P average value for each species and site was subsequently used in the analyses. Leaf material was oven dried at 70°C to constant mass. Dried leaf samples were ground into a fine powder using a ball mill (model MM 301, Retsch: Hann, Germany). Here, 3–4 mg of dried powder were weighed into tin capsules and then run through an Elemental analyser coupled to an Isotope Ratio Mass Spectrometer (20–22, Sercon Ltd, Crewe, UK) to determine leaf carbon (C) and nitrogen (N) concentration. Leaf P concentration was analysed through elements inductively coupled plasma mass spectrometry (VG101 analysis, ACME Analytical Laboratories: Vancouver, BC, Canada). Mass‐based N and P concentrations (N_m_ and P_m_; g g^−1^) and LMA were used to calculate the area‐based leaf N and P content (N_a_ and P_a_; g m^−2^). In addition, LMA was used to convert area‐based respiration (*R*_a_; µmol m^−2^ s^−1^) to a mass basis (*R*_m_; µmol g^−1^ s^−1^).

### Statistical analyses

The effect of temperature on *R*_d_ was tested using mixed‐effects ANOVA with site and successional group as main factors and species nested within successional groups as a random factor. The effect of drought on *R*_d_ for each species was analysed by a two‐way repeated measures ANOVA with month and site as main factors, and tree species as a random factor. The relationships between *R*_d_ (dependent variable) and *A*_n_, leaf N and P and LMA (independent variables) were analysed by a mixed‐effects linear regression with site as a main factor and species as a random factor. Effects were considered statistically significant at *P* < 0.05. Tukey's honest significance difference tests were used for post hoc comparisons. Residuals were visually checked for normality, and they were found to be normally distributed for all the parameters analysed. All analyses were performed in R (R Core Team, 2019). nlme R package (Pinheiro *et al*., 2019) was used to run the mixed‐effects ANOVA analyses, while, emmeans (Lenth, 2019) and multcomp (Hothorn *et al*., 2008) packages were used to run those post hoc tests.

## Results

**Fig. 1:**
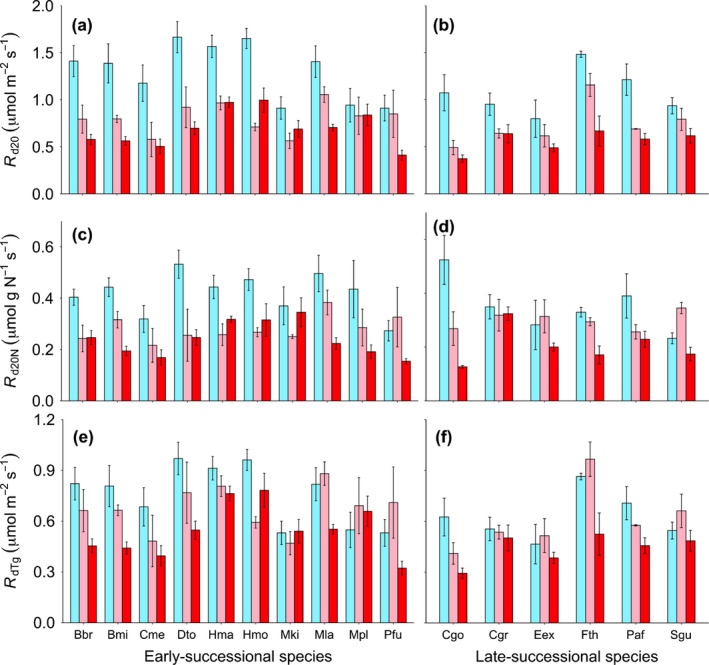
Leaf dark respiration measured during the wet season (November 2018) for 10 early‐successional (a, c, e) and six late‐successional (b, d, f) species. Leaf dark respiration at a common leaf temperature of 20°C (*R*_d20_, μmol m^−2^ s^−1^); *R*_d20_ normalised to total leaf N content (*R*_d20N_, μmol g N^−1^ s^−1^); leaf dark respiration at site‐specific nighttime growth temperature (*R*_gTg_, μmol m^−2^ s^−1^). Colours represent different sites (high‐elevation Sigira site, blue; mid‐elevation Rubona site, pink; low‐elevation Makera site, red). Means ± SE. *n* = 3–5. Abbreviations on *x*‐axis represent the 16 species (see full names in Table 2).

**Fig. 2:**
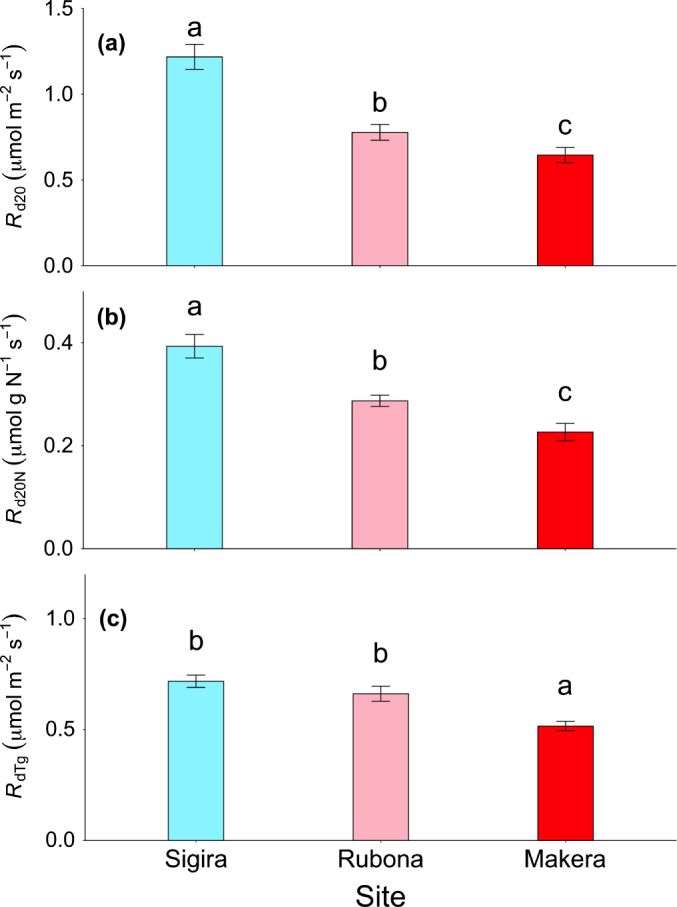
Leaf dark respiration measured during the wet season (November 2018) averaged across all 16 species at each site. Leaf dark respiration at a common leaf temperature of 20°C (*R*_d20_, μmol m^−2^ s^−1^) (a); *R*_d20_ normalised to total leaf N content (*R*_d20N_, μmol g N^−1^ s^−1^) (b); leaf dark respiration at site‐specific nighttime growth temperature (*R*_dTg_, μmol m^−2^ s^−1^) (c). Colours represent different sites (high‐elevation Sigira site, blue; mid‐elevation Rubona site, pink; low‐elevation Makera site, red). Means ± SE. *n* = 16. Different letters on bars represent differences across the three sites (Tukey post hoc test, *P* < 0.05).

Growth temperature strongly affected leaf dark respiration measured at a common leaf temperature of 20°C (*R*_d20_) such that it decreased with prevailing growth temperature (Figs 1a,b, 2a; Table 3). Compared with the high‐elevation site Sigira (1.23 ± 0.05 µmol m^−2^ s^−1^), *R*_d20_ averaged across all species was 36% lower at the mid‐elevation site Rubona and 47% lower at the low‐elevation site Makera. There was no significant difference between successional groups, neither in the magnitude of *R*_d20_ or in its acclimation capacity. In order to explore the interspecific variation in *R*_d20_, we ran a one‐way ANOVA at each site separately. There was a significant interspecific variation in *R*_d20_ at Sigira and Makera sites (*P* < 0.05), while no significant difference was detected at the Rubona site (*P* = 0.13).

**Table 3:** *P‐*values for effects of site, successional group, seasonal drought (hereafter called season) on leaf respiration

Mixed‐effects ANOVA			
	Site	Successional group	Site × successional group
*R*_d20_	**< 0.0001**	0.1	0.5
*R*_d20N_	**< 0.0001**	0.3	0.2
*R*_d20P_	**< 0.0001**	0.5	0.8
*R*_d20m_	**< 0.0001**	0.069	**0.024**
*R*_dTg_	**< 0.0001**	0.1	0.8

Mixed‐effects linear regression			
y‐variable	Trait	Site	Trait × Site
*R*_d20_	*A*_n_ **< 0.0001**	<** 0.0001**	**0.0014**
	N_a_ **< 0.0001**	**< 0.0001**	0.9
	P_a_ **0.0014**	**< 0.0001**	0.9
	LMA 0.8	**< 0.0001**	0.1

Repeated measures two‐way ANOVA			
Species	Season	Site	Site × Season
*Polyscias fulva*	**0.0063**	**0.018**	0.1
*Carapa grandiflora*	**0.009**	**0.0024**	**0.029**

Repeated measures three‐way ANOVA						
Season	Site	Species	Season × Site	Season × Species	Site × Species	Season × Site
**0.0005**	**0.0004**	**0.038**	**0.047**	0.055	0.46	0.7

Figures in bold indicate significant *P‐*values. *R*_d20_, leaf respiration on area basis; *R*_d20N_, leaf respiration normalised in leaf nitrogen; *R*_d20P_, leaf respiration normalised on leaf phosphorus; and *R*_d20m_, leaf respiration normalised at a common leaf temperature of 20°C; *R*_dTg_, leaf respiration estimated on the average nighttime air temperature of the month preceding the measurement campaign (i.e. October 2018).

We then normalised leaf *R*_d20_ to total leaf N, P and LMA in order to explore whether declines in leaf *R*_d20_ at mid‐elevation and low‐elevation sites were driven by concurrent decreases in these traits. Leaf *R*_d20_ normalised to leaf N (*R*_d20N_) was still lower at mid‐elevation and low‐elevation sites, being 25% and 41% lower at Rubona and Makera, respectively, compared with high‐elevation Sigira (0.39 ± 0.017 µmol g N^−1^ s^−1^; Figs 1c,d, 2b; Table 3). These effect sizes for acclimation are slightly smaller compared with those for area‐based *R*_d20_, due to somewhat lower values of area‐based leaf N (Supporting Information Fig. S1). However, they are still substantial, showing that only a small part of the site differences in *R*_d20_ can be attributed to variation in leaf N. Leaf *R*_d20_ normalised to leaf P (*R*_d20P_) was *c.* 35% lower at Makera compared with at Sigira (7.64 ± 0.64 µmol g P^−1^ s^−1^) but did not significantly differ between Sigira and Rubona sites (Fig. S2; Table 3). Lastly, also leaf *R*_d20_ normalised to LMA (*R*_d20m_) declined at mid‐elevation and low‐elevation sites (Fig. S3; Table 3). There was a significant site by successional group interaction for *R*_d20m_, reflecting lower values in LS compared with ES species in Sigira (32%) and Makera (29%), but rather similar values at Rubona site.

We also estimated *R*_d_ at nighttime growth temperature (*R*_dTg_) using site‐specific monthly average nighttime air temperatures, and a *Q*_10_ of 2.3. The average nighttime temperatures were 13.5, 17.8 and 17.1°c, for high‐elevation Sigira, mid‐elevation Rubona and low‐elevation Makera, respectively. Leaf *R*_dTg_ was 8% (nonsignificant) and 28% (significant) lower at Rubona and Makera sites, respectively, compared with Sigira (0.72 ± 0.027 µmol m^−2^ s^−1^; Figs 1e,f, 2c; Table 3). Similar *R*_dTg_ between Sigira and Rubona, but lower *R*_dTg_ at Makera meant that the strong thermal acclimation of *R*_d20_ resulted in homeostasis of *R*_dTg_ in trees at Rubona (i.e. similar *R*_dTg_) and overcompensation in trees at Makera (i.e. lower *R*_dTg_ compared with in Sigira). Our estimation of *R*_d_ at growth temperature was based on nighttime temperatures (as *R*_d_ was measured in darkness, after sunset), which were 0.9°C cooler in Makera compared with Rubona. If instead using 24 h mean temperatures, which were 0.45°C warmer in Makera compared with Rubona, the overcompensation in Makera would have been smaller (17%), but still significant.

As *Q*_10_ has also been shown to acclimate to warming in some studies (Atkin & Tjoelker, 2003), we performed a sensitivity analysis to check whether our conclusion changed when using growth temperature dependent and different *Q*_10_ values for each site. Values of *Q*_10_ calculated from Eqn 2 using site‐specific growth temperatures were 2.44, 2.23 and 2.21 for high‐elevation Sigira, mid‐elevation Rubona and low‐elevation Makera, respectively. These values resulted in leaf *R*_dTg_ being 4% (nonsignificant) and 25% (significant) lower at Rubona and Makera sites, respectively. Therefore, patterns of leaf *R*_dTg_ across sites were not changed significantly by the chosen *Q*_10_.

**Fig. 3:**
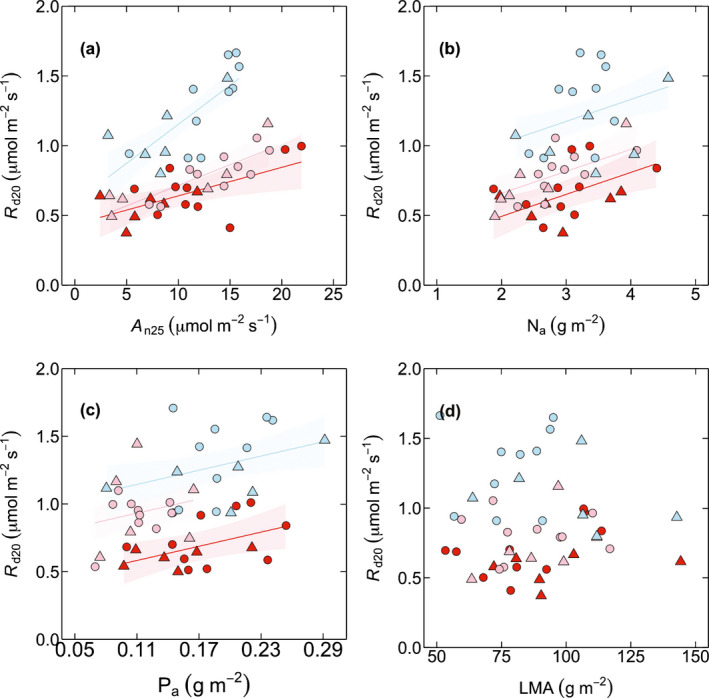
Leaf respiration measured at 20°C (*R*_d20_, μmol m^−2^ s^−1^) as a function of light saturated net photosynthesis at 25°C (*A*_n25_, μmol m^−2^ s^−1^; *R* ^2^ = 0.27, *R* ^2^ = 0.26 and *R* ^2^ = 0.21 for the high‐elevation Sigira site, mid‐elevation Rubona site and low‐elevation Makera site, respectively) (a), area‐based leaf nitrogen (N_a_, g m^−2^; *R* ^2^ = 0.037, *R* ^2^ = 0. 16 and *R* ^2^ = 0.097 for the high‐elevation Sigira site, mid‐elevation Rubona site and low‐elevation Makera site, respectively) (b), area‐based leaf phosphorus (P_a_, g m^−2^; *R* ^2^ = 0.045, *R* ^2^ = 0.11 and *R* ^2^ = 0.28 for the high‐elevation Sigira site, mid‐elevation Rubona site and low‐elevation Makera site, respectively) (c), and leaf mass per area (LMA, *R* ^2^ = 0.023, *R* ^2^ = 0.029 and *R* ^2^ = 0.068 for the high‐elevation Sigira site, mid‐elevation Rubona site and low‐elevation Makera site, respectively) (d). Symbols represent the successional groups (early‐successional species, circle; late‐successional species, triangle). Colours represent different sites (high‐elevation Sigira site, blue; mid‐elevation Rubona site, pink; low‐elevation Makera site, red). Each data point represents the average value of measured trees in each species (*n* = 3–5). (a–c) Shaded regions represent 95% confidence intervals of the regression line.

Across species there were positive relationships between leaf *R*_d20_ and net photosynthesis (*A*_n_), leaf N and P, but not with LMA (Fig. 3; Table 3). The positive relationship was strongest for *A*_n_ (adjusted *R* ^2^ = 0.78), compared with leaf N (adjusted *R* ^2^ = 0.62) and P (adjusted *R* ^2^ = 0.53). These *R* ^2^ represent the proportion of variations explained by the whole model that takes into account differences in intercepts across sites, as well as interactions in slopes when present. Moreover, while the slopes of these relationships were similar among sites for leaf N and P, they significantly differed for *A*_n_, declining from high‐elevation Sigira to low‐elevation Makera (Fig. 3a; Table 3). Nevertheless, residuals from the relationship between *A*_n_ and *R*_d20_ were not explained by variation in either leaf N or P (Fig. S4).

**Fig. 4:**
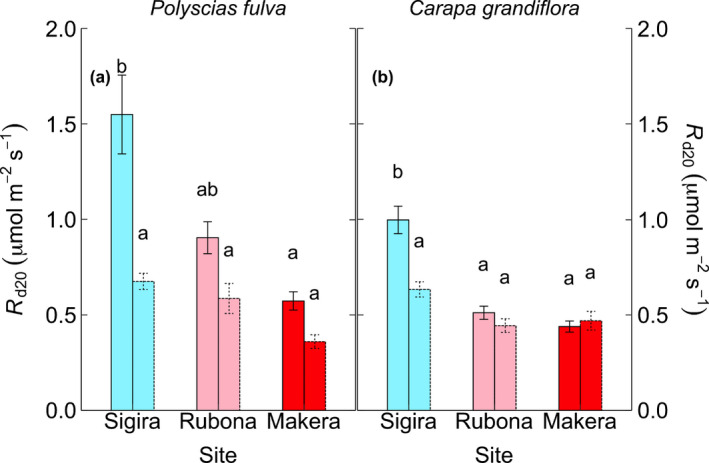
Leaf dark respiration measured at a common leaf temperature of 20°C (*R*_d20_, μmol m^−2^ s^−1^) at both early‐dry and late‐dry season in *Polyscias fulva* (an early‐successional species) (a) and *Carapa grandiflora* (a late‐successional species) (b). Bar lines represent measurement campaigns (early‐dry season, solid; late‐dry season, dashed). Colours represent different sites (high‐elevation Sigira site, blue; mid‐elevation Rubona site, pink; low‐elevation Makera site, red). Means ± SE. Different letters on bars represent differences across sites and measurement campaigns (Tukey’s post hoc test, *P < *0.05). *n* = 9 for Sigira site, and *n* = 5–6 for Rubona and Makera sites.

The *R*_d20_ of both *Polyscias fulva* and *Carapa grandiflora* was significantly more affected by the drought in high‐elevation Sigira compared with the mid‐elevation (Rubona) and low‐elevation (Makera) sites. The drought effect on *P. fulva* (ES species) was marginally stronger compared with *C. grandiflora* (LS species). Specifically, in *P. fulva*, the dry season reduced leaf *R*_d20_ by 56%, 36% and 37% at Sigira, Rubona and Makera sites, respectively (Fig. 4a; Table 3). In *C. grandiflora*, the dry season reduced *R*_d20_ by 36%, 14%, at Sigira and Rubona, respectively, while *R*_d20_ was slightly stimulated (7%) after the dry season in Makera (Fig. 4b; Table 3).

## Discussion

Previous studies have shown that leaf *R*_d_ at a given temperature is frequently downregulated in plants grown under warmer conditions, but typically not to an extent that prevents increased *R*_d_ at prevailing growth temperatures (Atkin & Tjoelker, 2003; Slot & Kitajima, 2015; Reich *et al*., 2016; Smith & Dukes, 2017). We showed here that thermal acclimation of *R*_d_ in African tropical trees was so strong (Fig. 1) that it led to either unchanged (at the intermediate elevation site) or lower values (at the lowest elevation site) compared with *R*_d_ at the highest and coolest site (Fig. 2). This finding of complete or even overcompensatory acclimation suggests that tropical tree species acclimate leaf *R*_d_ to warming more strongly compared with trees from temperate and boreal forests, which dominate previous studies (Slot & Kitajima, 2015; Reich *et al*., 2016; Smith & Dukes, 2017). This strong thermal acclimation of leaf *R*_d_ in tropical tree species compared with species in other biomes may be related to contrasting effects of warming on photosynthesis in trees from different biomes. Moderate warming usually stimulates photosynthesis more in temperate and boreal ecosystems compared with in tropical ecosystems (where it often declines) (Liang *et al*., 2013; Reich *et al*., 2018; Slot & Winter, 2018). This, in turn, is likely to be because tropical species are operating closer to their thermal optimum of net photosynthesis compared with temperate and boreal species (Huang *et al*., 2019).

Earlier studies of warming responses of *R*_d_ in tropical trees have indicated substantial, but partial, thermal acclimation (Cheesman & Winter, 2013a; Drake *et al*., 2015; Smith & Dukes, 2017; but see Scafaro *et al*., 2017). However, they were conducted on few species grown in controlled‐environment chambers and their field relevance is thus uncertain. The present study can draw more firm and general conclusions as it is based on 16 tropical species with different growth strategies grown freely rooted under field conditions. It should be recognised that elevation gradients are not perfect global warming proxies, as variables such as soil fertility, atmospheric pressure and radiation co‐vary with temperature (Malhi *et al*., 2010; Peng *et al*., 2020). At our experimental plantation sites, the soil water holding capacity differs, and was highest at the highest elevation site (Fig. S5). However, the measurements of *R*_d_ at a common temperature used to explore thermal acclimation were taken during the wet season, when soils were close to the site‐specific field capacity at all sites, to avoid confounding effects of soil moisture availability. Moreover, we have observed similar magnitudes of thermal acclimation of *R*_d_ in both a controlled‐environment chamber study (M. Wittemann *et al*., unpublished data) and a companion elevation gradient study with potted trees grown in the same soil (M. E. Dusenge *et al*., unpublished data); both using a subset of the species used in this study. We are therefore confident that our finding of (at least) complete thermal acclimation of R_d_ (i.e. *R*_d_ homeostasis at varying growth temperature) is likely to be a general response in African tropical highland trees. Moreover, we demonstrated, for the first time, that the response was equally strong in both ES and LS species (Fig. 1).

Our findings of substantial thermal *R*_d_ acclimation in tropical trees were in line with observational studies in the tropics, showing that leaf *R*_d_ at a common temperature is usually higher in trees growing in a cool, montane climate compared with trees in warmer, lowland forests (Meir *et al*., 2001; Cavaleri *et al*., 2008; van de Weg *et al*., 2012; Rowland *et al*., 2017). This pattern may reflect, at least to some extent, a thermal acclimation response of leaf *R*_d_. However, the possible acclimation response is indistinguishable from effects of species adaptations to these contrasting thermal environments, as well as from variation in soil fertility across different sites.

So far, there is still no clear consensus regarding the exact biochemical and physiological mechanisms underpinning thermal acclimation of leaf *R*_d_ (Dusenge *et al*., 2019). Some studies have shown that thermal acclimation of leaf *R*_d_ is mediated by photosynthesis (e.g. Dusenge *et al*., 2019; Wang *et al*., 2020). Based on optimality theory, Wang *et al*. (2020) suggested that thermal acclimation of leaf *R*_d_ is strongly driven by concomitant thermal acclimation of *V*_cmax_. In order to test to what extent this theory may explain the observed thermal acclimation of leaf *R*_d_, we derived *V*_cmax_ (at 25°C) from net photosynthesis data using the ‘one‐point method’ (De Kauwe *et al*., 2016). We then determined one *V*_cmax_ value for each successional group at each site using the average value of species in each group. Using the proposed theoretical response of both leaf *R*_d_ and *V*_cmax_ at a common temperature (Table 1 in Wang *et al*., 2020), we calculated the predicted shifts in both leaf *R*_d_ (at 20°C) and *V*_cmax_ (at 25°C) for all three sites. The predicted reductions in *R*_d20_ were, on average, 29.1% and 28.5% against 36% and 47% of observed reduction for the mid‐elevation Rubona and low‐elevation Makera, respectively. For *V*_cmax25_, the model predicted a reduction of 14% and 28%, while we observed an increase of 5% and a reduction of 12% for the mid‐elevation Rubona and low‐elevation Makera, respectively. This shows that the strong thermal acclimation of leaf R_d_ observed in our study was not in agreement with the optimality model, which predicted more moderate (partial) acclimation. The reason for this is unclear, and hard to determine. It is possible that stomatal conductance, and thus net photosynthesis, responded more negatively to increasing vapour pressure deficit at lower elevation (Table 2) than predicted by optimality or combined stomatal‐photosynthesis models. This may particularly be the case if leaf temperatures are considerably higher than air temperatures (used in the model), as observed for some of our species in previous studies (Vårhammar *et al*., 2015; Ntawuhiganayo *et al*., 2020). In addition, radiation is higher at the two lower elevation sites (Table 2), with uncertain net effects on photosynthesis (stimulating photosynthesis but at the same time increasing leaf‐to‐air vapour pressure deficit and thereby increasing stomatal limitations of photosynthesis).

We normalised leaf *R*_d_ by leaf N and P, and LMA to explore to what extent thermal acclimation was mediated by these traits. It was not, since *R*_d_ expressed per unit leaf N, P or mass was still lower in warm‐grown trees (Figs 2, S2, S3). This implies that other traits or mechanisms caused the observed thermal acclimation. It is possible that shifts in within‐cell anatomy or biochemistry may be involved. For example, previous studies have shown that lower leaf *R*_d_ in warm‐grown plants correlated with declines in mitochondrial density (Armstrong *et al*., 2006), cytochrome *c* oxidase (COX) (Rashid *et al*., 2020), and some intermediates of glycolysis and tricarboxylic acid cycle (Ahmad Rashid *et al*., 2020).

As also observed in our study (Fig. 3), tree and species variation in leaf R_d_ has been linked with other more commonly measured leaf traits. These traits include *A*_n_ (Hoefnagel *et al*., 1998; Atkin *et al*., 2005; Crous *et al*., 2017a; Rowland *et al*., 2017; O'Leary *et al*., 2019) as well as leaf N (Ryan, 1995; Reich *et al*., 1998; Wright *et al*., 2006; Atkin *et al*., 2005; Crous *et al*., 2017a; Rowland *et al*., 2017) and P (Theodorou & Plaxton, 1993; Meir *et al*., 2001; Wang *et al*., 2015; Crous *et al*., 2017a; Rowland *et al*., 2017) LMA has also been shown to be positively related to leaf R_d_ in other studies (e.g. Reich *et al*., 1998; Meir *et al*., 2001; Wright *et al*., 2006; Crous *et al*., 2017a; Rowland *et al*., 2017), although this was not the case in our study (Fig. 3d). In the present study, *A*_n_ was the strongest predictor, with leaf N and P adding no further explanatory power (Fig. S4). This strong dependence of leaf *R*_d_ on photosynthesis is probably driven by carbohydrate supply from photosynthesis, which is the main source of substrates for respiration metabolism (Griffin *et al*., 2002; Whitehead *et al*., 2004; O’Leary *et al*., 2017, 2019).

### Effect of dry season on leaf R_d_

Leaf *R*_d_ measured at 20°C (*R*_d20_) was reduced at the end of seasonal drought in the two studied species, and this reduction was strongest in trees at the coolest high‐elevation site and in *Polyscias fulva*, the ES species (Fig. 4). These results partly contrast with preliminary analyses of net photosynthesis from the August campaign (M. Mujawamariya *et al*., unpublished data). Net photosynthesis decreased strongly during the late‐dry season at the mid‐elevation and low‐elevation sites, but not at Sigira where *R*_d20_ decreased the most. Moreover, as opposed to *R*_d_ results, net photosynthesis exhibited similar responses in both species. This means that photosynthesis data did not help to explain either species or site differences in the dry season response of *R*_d_.

Site differences in *R*_d20_ were large at the beginning of the dry season but similar across sites towards the end of the dry season. For *R*_d_ at prevailing nighttime temperatures, this implies rather similar rates under nondrought conditions but increased *R*_d_ at warmer and drier sites late during the dry season. This could reflect an increased need for maintenance respiration to support hydraulic repair at sites with more pronounced drought, as indicated in previous studies (Atkin & Macherel, 2009; Brodersen & McElrone, 2013; Rowland *et al*., 2015b).

Many Earth System Models (ESMs) do not incorporate thermal acclimation of autotrophic respiration when predicting terrestrial carbon exchange in future climates (Smith & Dukes, 2013; Atkin *et al*., 2015; Huntingford *et al*., 2017). Accurate representation of thermal acclimation of leaf *R*_d_ requires proper formulation of equations that are, at least, specific to each biome and plant functional type (Atkin *et al*., 2015; Smith *et al*., 2015). In particular, the representation of thermal acclimation of leaf *R*_d_ for the tropical biome in ESMs has been hindered by a paucity of data on this process (Vanderwel *et al*., 2015). The present study reduces this knowledge gap, and suggests that tropical trees in the field acclimate more strongly to rising growth temperature compared with most tree species from other biomes (e.g. Reich *et al*., 2016), and that successional groups respond similarly.

### Conclusion

We demonstrated strong downregulation of leaf *R*_d_ at lowest and intermediate elevation sites across 16 tropical tree species grown along an elevation gradient. The reduction in *R*_d_ measured at 20°C was so strong that values of *R*_d_ at prevailing nighttime growth temperatures were either unchanged (at the intermediate elevation site) or even lower (at the lowest elevation site) compared with *R*_d_ at the highest and coolest site. Downregulation of *R*_d_ was equally strong in species with early‐successional and late‐successional strategies. The strong thermal acclimation of *R*_d_ could not be explained by shifts in leaf N_a_, P_a_ or LMA and was stronger compared with expected values based on *A*_n_, data. However, differences in *A*_n_ among species accounted for a large share of the interspecific variation in *R*_d_, with leaf nutrients and LMA not contributing additional explanatory power. Furthermore, we showed that drought reduced *R*_d_ and this effect differed between both species and sites. Our findings show that the strong thermal acclimation of leaf *R*_d_ in tropical tree species should be accounted for in order to avoid overestimation of the impact of global warming on autotropic respiration in tropical forests.

## Author contributions

MM, GW and JU conceived and designed the study with important contributions from DN; MW, MM and AM conducted field measurements; MM, JU and MED analysed the data and wrote the manuscript with important contribution from GW; MW, AM, BN, EZ, DN provided editorial advice.

## Supporting information


**Fig. S1** Area‐based leaf nitrogen (N_a_, g m^−2^) during the wet season (November 2018) for 10 early‐successional and six late‐successional species, and averaged across species at each site.
**Fig. S2** Leaf dark respiration measured at a common leaf temperature of 20°C and normalised to leaf P (*R*_d20P_, μmol g P^−1^ s^−1^) during the wet season (November 2018) and averaged across the 16 species at each site.
**Fig. S3** Leaf dark respiration measured at a common leaf temperature of 20°C and normalised to LMA (*R*_d20m_, μmol g^−1^ s^−1^) during the wet season (November 2018) for 10 early‐successional and six late‐successional species, and averaged across species and successional group at each site.
**Fig. S4** Residuals from the relationship between *A*n and *R*d20 as a function of leaf N (N_a_, g m^−2^) (a) and leaf P (P_a_, g m^−2^).
**Fig. S5** Soil water content at 0–20 cm soil depth during November 2018 and the second half of April 2019 measured by six sensors at each site.
Please note: Wiley Blackwell are not responsible for the content or functionality of any Supporting Information supplied by the authors. Any queries (other than missing material) should be directed to the *New Phytologist* Central Office.
