# Supplementary material for: Complete or overcompensatory thermal acclimation of leaf dark respiration in African tropical trees
Source: New Phytol. 2020 Dec 6;229(5):2548–61. doi: 10.1111/nph.17038 (PMC7898918; doi:10.1111/nph.17038)
Supplement: Supplementary file 1 — Fig. S1 Area‐based leaf nitrogen (Na, g m−2) during the wet season (November 2018) for 10 early‐successional and six late‐successional species, and averaged across species at each site. Fig. S2 Leaf dark respiration measured at a common leaf temperature of 20°C and normalised to leaf P (R d20P, μmol g P−1 s−1) during the wet season (November 2018) and averaged across the 16 species at each site. Fig. S3 Leaf dark respiration measured at a common leaf temperature of 20°C and normalised to LMA (R d20m, μmol g−1 s−1) during the wet season (November 2018) for 10 early‐successional and six late‐successional species, and averaged across species and successional group at each site. Fig. S4 Residuals from the relationship between An and Rd20 as a function of leaf N (Na, g m−2) (a) and leaf P (Pa, g m−2). Fig. S5 Soil water content at 0–20 cm soil depth during November 2018 and the second half of April 2019 measured by six sensors at each site. Please note: Wiley Blackwell are not responsible for the content or functionality of any Supporting Information supplied by the authors. Any queries (other than missing material) should be directed to the New Phytologist Central Office. [file NPH-229-2548-s001.pdf]

New Phytologist

***New Phytologist* Supporting Information**

Article title: **Complete or over-compensatory thermal acclimation of leaf dark respiration in African tropical trees**

Authors: [1,2]Myriam Mujawamariya, [1,2]Maria Wittemann, [1,2]Aloysie Manishimwe, [1,2,3]Bonaventure Ntirugulirwa, [1]Etienne Zibera, [4]Donat Nsabimana, [2]Göran Wallin, [2]Johan Uddling, [1,2]Mirindi Eric Dusenge

[1]Department of Biology, University of Rwanda, University Avenue, P.O. Box 117, Huye, Rwanda

[2]Department of Biological and Environmental Sciences, University of Gothenburg, P.O. Box 461, SE-405 30 Gothenburg, Sweden

[3]Rwanda Agriculture and Animal Development Board, P.O. Box 5016, Kigali, Rwanda

[4]School of Forestry and Biodiversity and Biological Sciences, University of Rwanda, Busogo, Rwanda

Correspondence: johan.uddling@bioenv.gu.se

Article acceptance date: 16 October 2020

New Phytologist

The following Supporting Information is available for this article:

Fig. S1 Area-based leaf nitrogen (N_a, g m^{-2}) during the wet season (November 2018) for ten early-successional (a) and six late-successional (b) species (a), and averaged across species at each site (c).

Fig. S2 Leaf dark respiration measured at a common leaf temperature of 20°C and normalized to leaf P (R_{d20P}, µmol g P^{-1} s^{-1}) during the wet season (November 2018) and averaged across the 16 species at each site.

Fig. S3 Leaf dark respiration measured at a common leaf temperature of 20°C and normalized to LMA (R_{d20m}, µmol g^{-1} s^{-1}) during the wet season (November 2018) for ten early-successional (a) and six late-successional (b) species, and averaged across species and successional group at each site (c).

Fig. S4 Residuals from the relationship between A_n and R_{d20} as a function of leaf N (N_a, g m^{-2}) (a) and leaf P (P_a, g m^{-2}).

Fig. S5 Soil water content at 0-20cm soil depth during November 2018 and the second half of April 2019 measured by six sensors at each site.

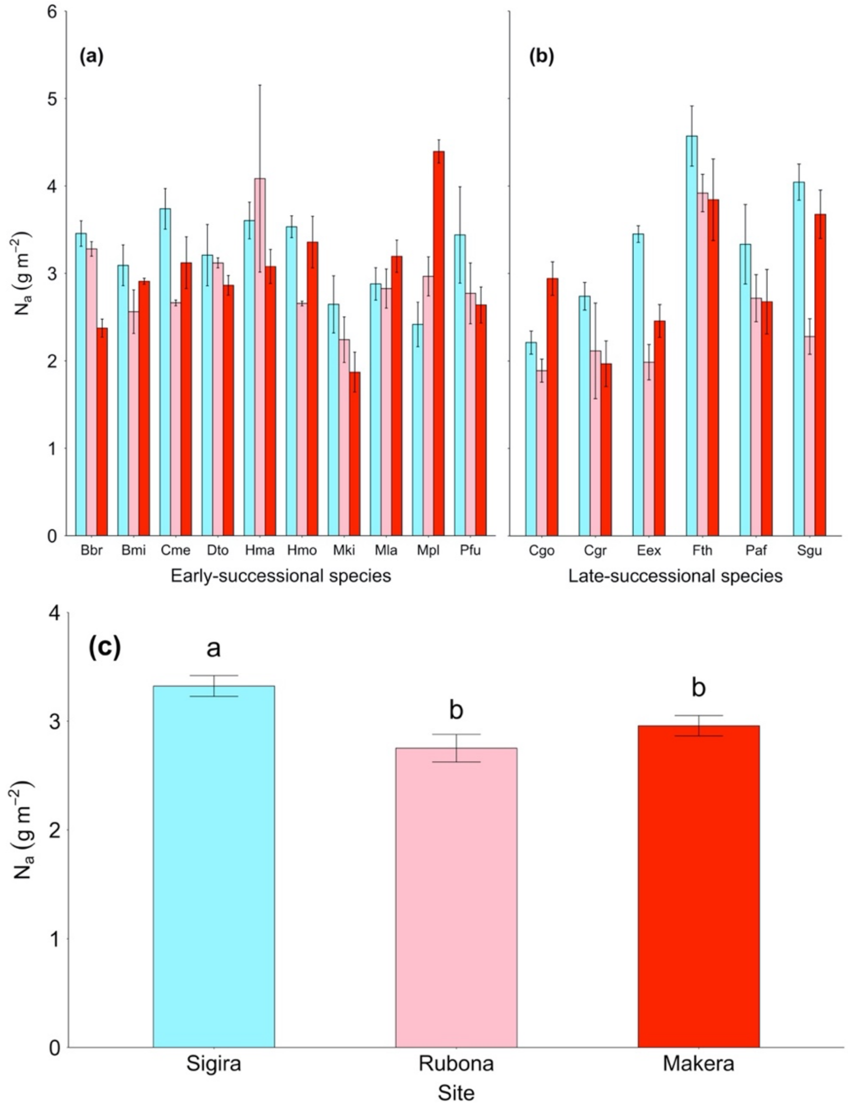

Fig. S1 Area-based leaf nitrogen (N_a, g m^{-2}) during the wet season (November 2018) for ten early-successional (a) and six late-successional (b) species (a), and averaged across species at each site (c). Colors represent different sites (high-elevation Sigira site = blue; mid-elevation Rubona site = pink; low-elevation Makera site = red). Means ± *SE*. Different letters on bars in (c) represent differences across sites (Tukey post hoc test, $p < 0.05$). n = 3-5 for (a, b) and n = 16 for (c). Abbreviations on x-axis in (a, b) represent the 16 species (See full names in Table 2).

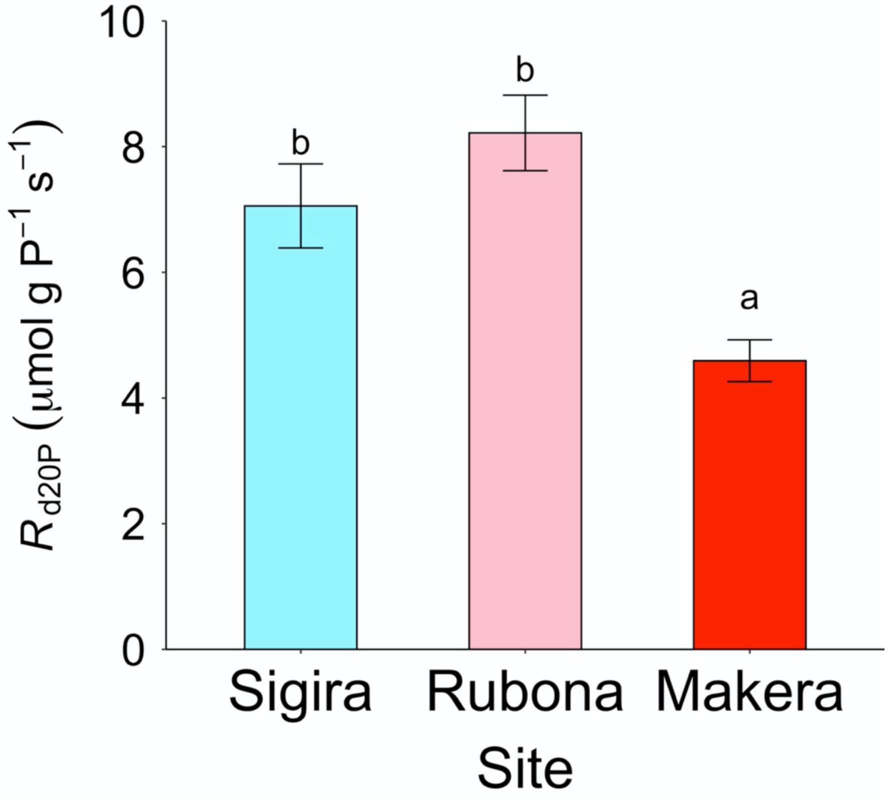

Fig. S2 Leaf dark respiration measured at a common leaf temperature of 20°C and normalized to leaf P (R_{d20P}, μmol g P^{-1} s^{-1}) during the wet season (November 2018) and averaged across the 16 species at each site. Colors represent different sites (high-elevation Sigira site = blue; mid-elevation Rubona site = pink; low-elevation Makera site = red). Means ± *SE*. Different letters on bars in (b) represent differences across sites (Tukey post hoc test, $p < 0.05$). n = 16.

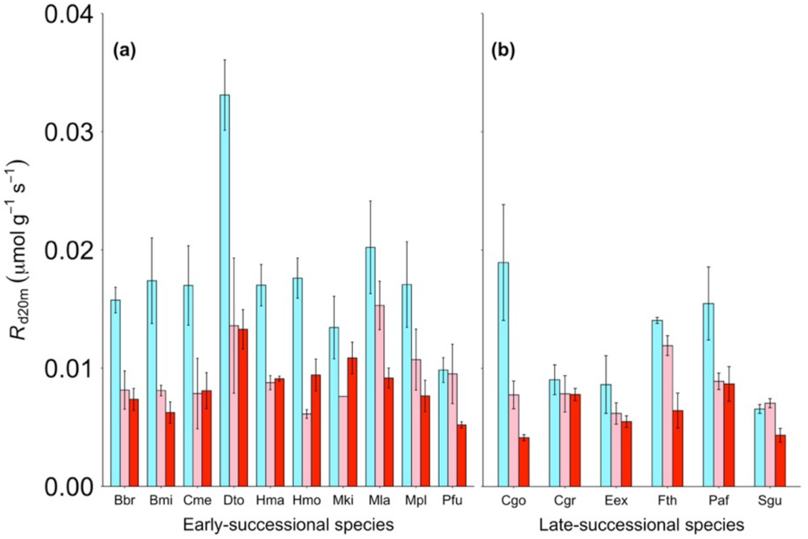

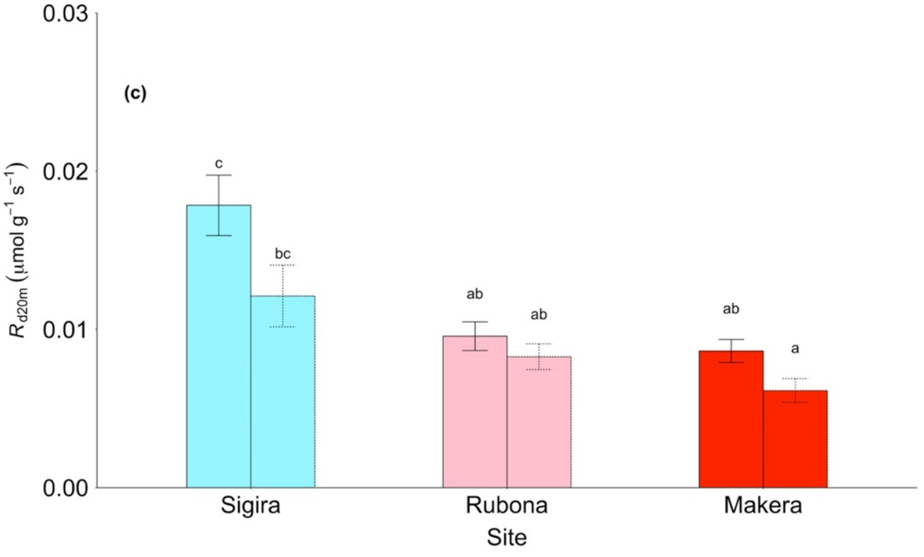

Fig. S3 Leaf dark respiration measured at a common leaf temperature of 20°C and normalized to LMA (R_{d20m}, µmol g^{-1} s^{-1}) during the wet season (November 2018) for ten early-successional (a) and six late-successional (b) species, and averaged across species and successional group at each site (c). Colors represent different sites (high-elevation Sigira site = blue; mid-elevation Rubona site = pink; low-elevation Makera site = red). Means ± *SE*. Different letters on bars in (c) represent differences across sites (Tukey post hoc test, $p < 0.05$). n = 3-5 for (a, b) and n = 16 for (c). Abbreviations on x-axis represent the 16 species (See full names in Table 2).

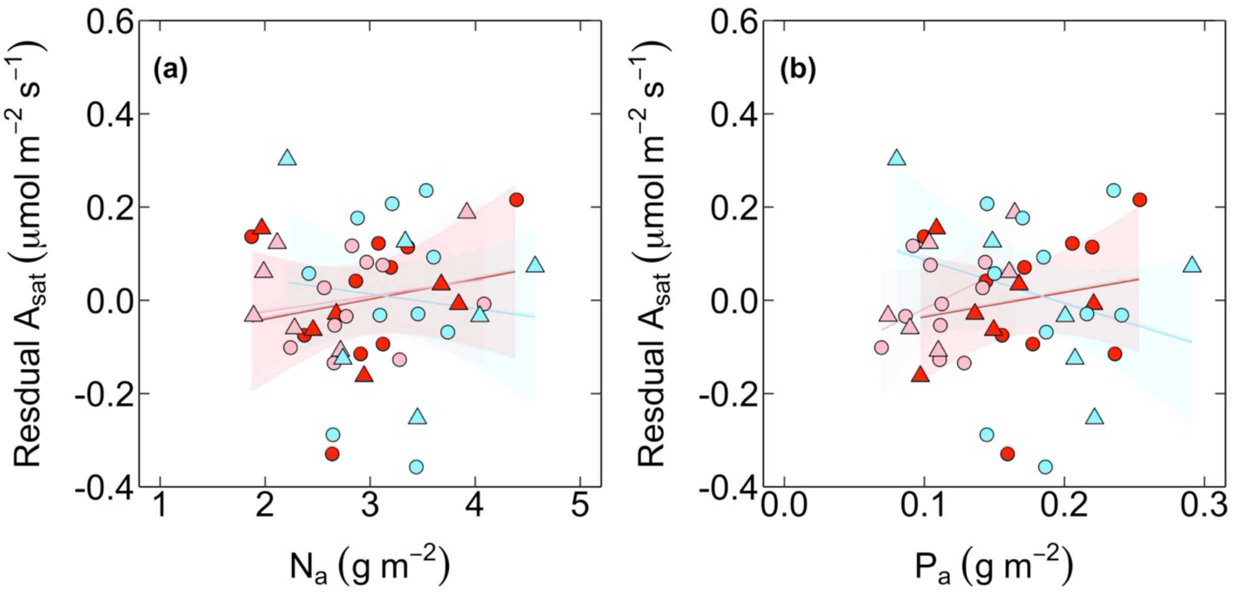

Fig. S4 Residuals from the relationship between A_n and R_{d20} as a function of leaf N (N_a, g m^{-2}) (a) and leaf P (P_a, g m^{-2}). Symbols represent the successional groups (early-successional species = circle; late-successional species = triangle). Colors represent different sites (high-elevation Sigira site = blue; mid-elevation Rubona site = pink; low-elevation Makera site = red). Each data point represents the average value of measured trees in each species (n = 3-5). Shaded regions represent 95% confidence intervals for the regression lines.

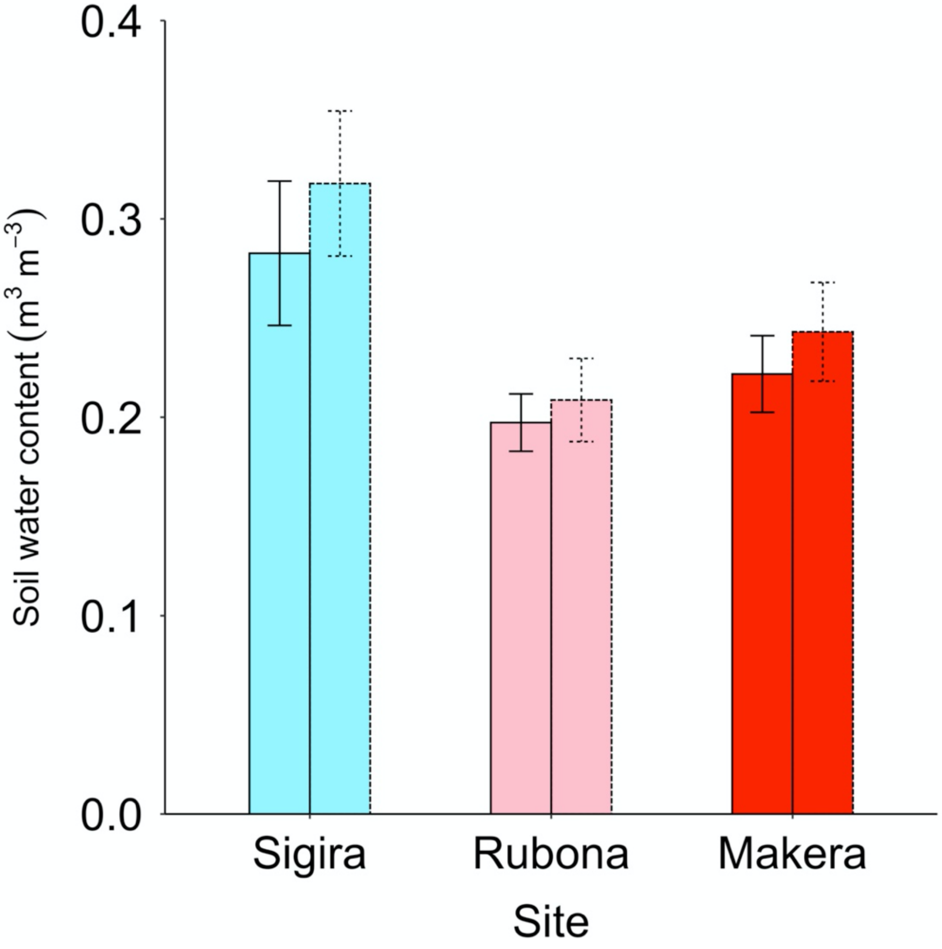

Fig. S5 Soil water content at 0-20cm soil depth during November 2018 and the second half of April 2019 measured by six sensors at each site. Medians±*SE*. Medians are calculated for each sensor and averaged across all six sensors. Error bars indicate standard error among different sensors ($n = 6$). The April data represent the site-specific field capacity as they were measured during the late part of the annual main rainy season (normally from March through May, but ending a bit into May in the lowest elevation site). Color represents different sites (blue = high-elevation Sigira; pink = mid-elevation Rubona; red = low-elevation Makera). Line shape for bars and corresponding error bars represent different rainfall periods (solid = November 2018, short-rainy season; dashed = April 2019, highest precipitation period).
